# Breaking Immunosuppression to Enhance Cancer Stem Cell-Targeted Immunotherapy

**DOI:** 10.7150/ijbs.101025

**Published:** 2025-02-10

**Authors:** Fang Zheng, Shan Zhang, Alfred E. Chang, James J. Moon, Max S. Wicha, Shelley Xuelai Wang, Junhui Chen, Jixian Liu, Fanjun Cheng, Qiao Li

**Affiliations:** 1 Department of Pediatrics, Union Hospital, Tongji Medical College, Huazhong University of Science and Technology, Wuhan 430022, Hubei Province, China.; 2 Institute of Hematology, Union Hospital, Tongji Medical College, Huazhong University of Science and Technology, Wuhan 430022, Hubei Province, China.; 3 Department of Surgery, University of Michigan, Ann Arbor, Michigan 48109, USA.; 4 Department of Pharmaceutical Sciences, University of Michigan, Ann Arbor, Michigan 48109, USA.; 5 Department of Internal Medicine, University of Michigan, Ann Arbor, Michigan 48109, USA.; 6 Asian Academy of Aging Research & Translational Medicine, Shenzhen, China.; 7 Peking University Shenzhen Hospital, Shenzhen, China.

**Keywords:** cancer stem cells, immunotherapy, immunosuppression, cancer vaccines, bispecific antibodies, antibody drug conjugates

## Abstract

Cancer stem cell (CSC)-targeted immunotherapy has emerged as a novel strategy in cancer treatment in the past decade. However, its efficacy is significantly limited due to the existence of host immune suppressive activity. Specifically, programmed cell death ligand-1 (PD-L1) is overexpressed in CSCs, and PD-L1 overexpressed CSCs create immunosuppressive milieu *via* interacting with various immune cells in tumor microenvironments (TME). Hence, novel immunotherapeutic strategies targeting CSCs with concurrent immunosuppression interruption will be promising in enhancing anti-CSC effects. These include dendritic cell (DC) and nanodisc (ND)-based vaccines to present CSC antigens in the forms of CSC lysate, CSC-marker proteins, and CSC-derived peptides to induce anti-CSC immunity. In addition, CSC-directed bispecific antibodies (BiAbs) and antibody drug conjugates (ADCs) have been developed to target CSCs effectively. Furthermore, chimeric antigen receptor (CAR)-T cell therapy and natural killer (NK) cell-based therapy targeting CSCs have achieved progress in both solid and hematologic tumors, and inhibition of CSC associated signaling pathways has proven successful. In this review, we aimed to outline the roles and regulatory mechanisms of PD-L1 in the properties of CSCs; the crosstalk between CSCs and immunosuppressive cells in TME, and recent progress and future promises of immunosuppression blockage to enhance CSC-targeted immunotherapy.

## Background

The concepts and clinical practices of blocking the interactions between tumor surface programmed cell death ligand-1 (PD-L1) and the programmed cell death protein 1 (PD-1) on the surface of effector T cells have revolutionized cancer immunotherapies. Anti-PD-1/PD-L1 therapies have become the standard therapy in many cancer types, including melanoma, non-small cell lung cancer, colorectal cancer, triple-negative breast cancer, head and neck squamous cell carcinoma, and hepatocellular carcinoma 1. Despite advances in this field, only a minority of patients demonstrated benefits with anti-PD-1/PD-L1 therapy. Currently limitations of PD-L1 antibodies are mainly due to certain drawbacks such as tumor hyperprogression 2; poor bioavailability; high cost and complicated processing, and immunogenic adverse reactions 3. Increasing evidence indicates that PD-L1 is found not only on the tumor cell membrane but also within the cytoplasm, exosomes, and even nucleus. Based on these, it has been reported that the dynamic and spatial heterogeneous expression of PD-L1 in tumors is mainly responsible for the unsatisfactory efficacy of PD-L1 antibodies 4. In addition, previous studies have demonstrated increased expression of PD-L1 in CSCs of multiple cancers, resulting in more aggressive resistance of CSCs to anti-PD-1/PD-L1 therapies, and CSC-intrinsic PD-L1 pathways take a nonnegligible role in cancer progression and metastasis in an immune independent way 5-11. Intrinsic and acquired resistance to these agents represents a significant obstacle in their clinical application 12.

One putative theory for resistance to anti-PD-1/PD-L1 therapy was the existence of cancer stem cells (CSCs) in heterogeneous tumor microenvironment (TME). CSCs could be identified by surface protein markers; intracellular molecular signature including aldehyde dehydrogenase (ALDH) activity; stemness-related signaling pathways; drug carriers, and dynamic states of metabolome and cellular differentiation 13. Tumor initiation, progression, metastasis, relapse, and therapeutic resistance endowed by CSCs make them vital targets for cancer immunotherapy. Moreover, the interactions of CSCs with multiple immunosuppressive cells such as myeloid-derived suppressor cells (MDSCs), tumor-associated macrophages (TAMs), regulatory dendritic cells (DCregs) and regulatory T cells (Tregs) in the TME further decrease the effectiveness of cancer immunotherapy 14.

Previous studies have demonstrated increased expression of PD-L1 in CSCs of multiple cancers such as non-small cell lung cancer, hepatocellular carcinoma, breast cancer, and colorectal cancer 5-10, which may partly explain more pronounced immune evasion features of the CSCs. Consequently, PD-L1-overexpressing CSCs create an immunosuppressive milieu by cooperating with other noncancerous cells in the TME 11. Furthermore, CSC-intrinsic PD-L1 pathways take a nonnegligible role in cancer progression and metastasis in an immune independent way, adding more complexity to the mixed response of cancer patients to PD-1/PD-L1 blockade. PD-1/PD-L1 molecules also showed various expression on different types of immune cells in TME, exerting complicated impacts on the communications between the immune cells and CSCs. In this regard, concurrent CSC-targeted therapy with PD-1/PD-L1 blockage might lead to the development of more promising strategy in cancer immunotherapy. In this review, we aimed to outline the roles and regulatory mechanisms of PD-L1 in the properties of CSCs; the crosstalk between CSCs and immunosuppressive cells in TME; and the promise of PD-1/PD-L1 blockage to enhance CSC-targeted immunotherapies.

## The roles and regulation of PD-L1 in CSCs

### Characteristics of CSCs

To date, various markers have been used characterize CSCs in different cancer types, e.g., CD24, CD44, CD133, ALDH, ABCB5, EpCAM, LGR5, etc. CSCs are capable of proliferating extensively with a self-renewal ability, which enables them to initiate tumors in both immunocompromised and immunocompetent hosts 15, 16. In contrast, xenotransplantation of non-CSCs into highly immunocompromised NSG mice resulted in tumor formation but not in immunocompetent models. These suggest that immune evasion represents a fundamental feature of CSCs. Antigen processing and presentation are impaired in CSCs as a result of low major histocompatibility complex (MHC) molecule expression as well as downregulation of other antigen processing molecules 17, 18. In addition, downregulation of tumor-associated antigens (TAAs) was observed in CSCs, leading to inefficient recognition of CSCs by dendritic cells (DCs), which in turn resulted in reduced activation of T cells 19. Compared with non-CSCs, CSCs retain a more intact DNA repair function that reduces immunogenicity. CSCs preferentially secrete various immune factors at higher levels than non-CSCs such as transforming growth factor β (TGF-β), IL-6, IL-1β and IL-10 in breast 20, 21, glioma 22, hepatic 23 and pancreatic 24 CSCs. These cytokines underlay CSC maintenance and immune suppression. Another clinically relevant property of CSCs is their resistance to traditional chemotherapies and radiotherapies. Unlike non-CSCs, CSCs tend to stay in dormant or quiescent state, protecting them from cytotoxic drugs targeting rapidly proliferating cancer cells 25. High expression of efflux transporters and anti-apoptotic proteins and the presence of reactive oxygen species scavengers are associated with CSC drug resistance 26. Development of strategies to target CSCs based on their distinct biological characters thus represents promising efforts in cancer immunotherapy.

### PD-L1 expression in CSCs

**Figure 1:**
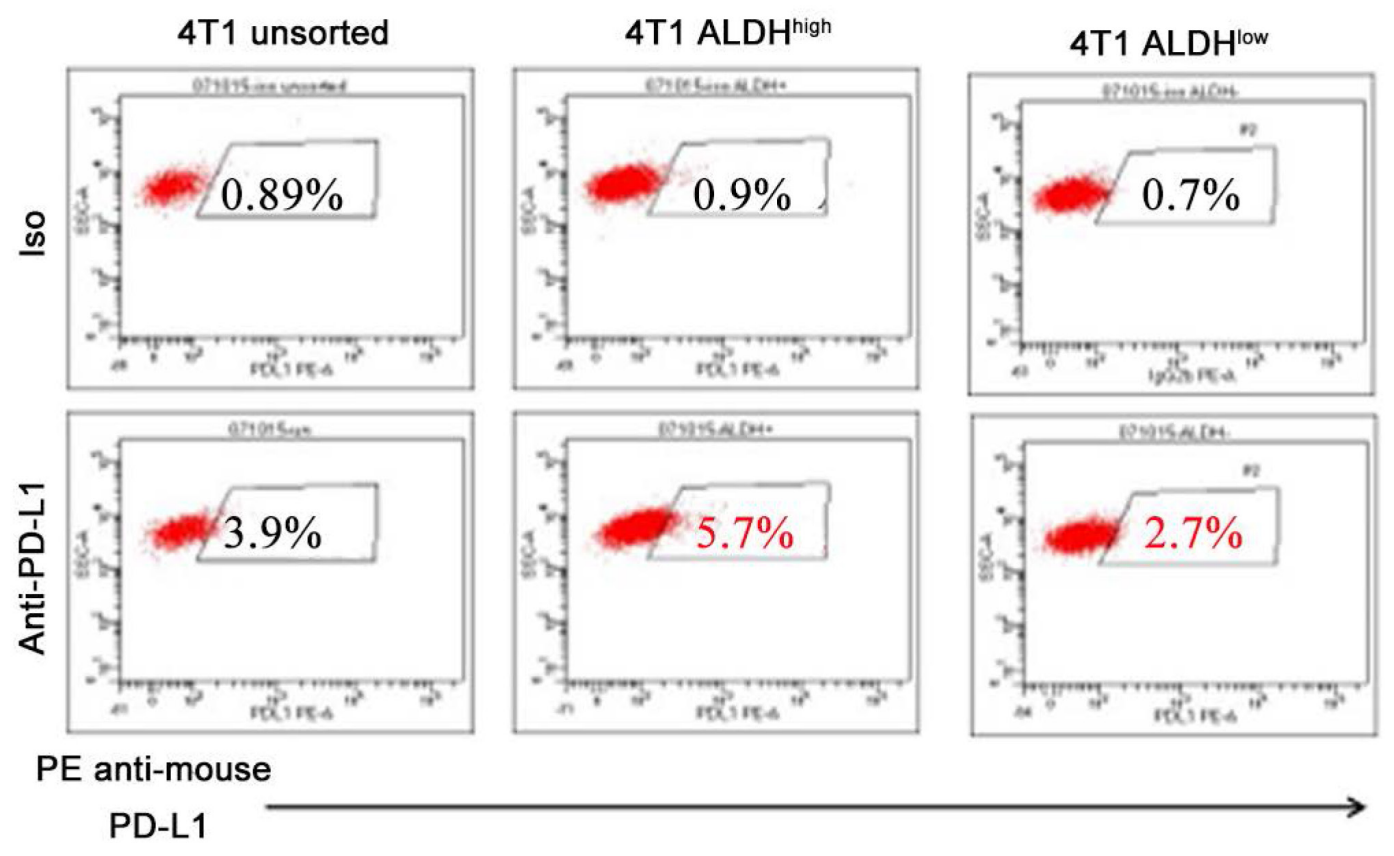
Expression of PD-L1 is higher on 4T1 ALDH^high^ CSCs than on 4T1 ALDH^low^ non-CSCs. These figures represent flow cytometry assessments conducted by three members of our lab. Our lab, as well as many others, have successfully used the ALDEFLUOR Kit (StemCell Technologies) to isolate ALDH^high^ vs ALDH^low^ cells 89, 93, 98. Most importantly, we determined the stemness of ALDH^high^ cells in our previous studies 89, 93, 110.

Accumulating literatures have illustrated that PD-L1 is highly expressed in CSCs in solid tumors, including hepatocellular carcinoma 5, gastric cancer 27, lung cancer 28, breast and colon cancer 29, pancreatic cancer 11, and melanoma 30 (**Table 1**). ALDH has been wildly used as a marker for CSCs. We observed that the expression levels of PD-L1 in ALDH^high^ CSCs were significantly elevated, compared with those in ALDH^low^ non-CSCs by more than twofolds in murine breast cancer cell line (**Figure 1**). In endometrial carcinoma, high expression of PD-L1 in CD133^+^ CSCs was observed and found associated with stemness markers ALDH, OCT4, SOX2, NANOG 31. In breast cancer and malignant mesothelioma, PD-L1 expression is also positively correlated with CSC markers such as CD44, ALDH, ALCAM 32, 33. It was reported that high PD-L1 expression in pancreatic cancer tissues with high levels of CD44^+^/CD133^+^ CSCs predicts an unfavorable prognosis of pancreatic cancer 11. It was also reported that high PD-L1 expression was strongly associated with high expression of the stemness markers CD44 and LGR5 in ovarian cancer 34. These observations have suggested the prognostic value of PD-L1 expression in CSCs and emphasized the significance of targeting PD-L1^high^ CSCs.

### Intrinsic roles and regulation mechanisms of PD-L1 in CSCs

Although the interaction between tumor cell PD-L1 and T cell PD-1 has been widely characterized, the mechanisms regulating PD-L1 expression in cancers have been reviewed by Yamaguchi H, *et al.* 35. However, the intrinsic role of PD-L1 in CSCs and its expression regulation are less defined. Notch3 was found to be overexpressed in PD-L1^high^ breast cancer cells with the nature of stemness. The effect of Notch inhibitors on PD-L1 overexpression in CSCs was completely abrogated upon mTOR knockdown, demonstrating that overexpression of PD-L1 in CSCs was at least partly mediated by the Notch pathway through Notch3/mTOR axis 8. CSC-intrinsic PD-L1 expression is also regulated *via* the PI3K/AKT/mTOR pathways as evidenced by the fact that the mTOR inhibition *via* Rapamycin decreased the cancer cell stemness in a similar fashion to PD-L1 knock down 36. The transcription factors HIF-1α and HIF-2α activation triggered high level PD-L1 expression, which correlated with modulated self-renewal and tumorigenicity of CSCs 16. It was also established that PD-L1 interacts with Frizzled 6 to activate β-catenin and form a positive feedback loop to promote CSC maintenance and expansion 37. Epithelial-mesenchymal transition (EMT) is essential for enhanced tumor migratory and invasive capabilities. Previous studies reported that PD-L1 promotes EMT features and cancer cell stemness, and EMT enriches PD-L1 in CSCs by the EMT/β-catenin/STT3/PD-L1 signaling axis 6, 7. From the perspective of epigenetically regulation, CSC-intrinsic PD-L1 is regulated by histone modifications 38. Post-transcriptionally modulation of PD-L1 expression by microRNA is also found to regulate breast cancer cell stemness 39. The direct binding of miR-873 to 3′-untranslated regions (UTR) of PD-L1 inhibited its expression, thus attenuated the stemness and chemoresistance of breast cancer cells, which depended on the downstream PI3K/Akt and ERK1/2 signaling pathways. Furthermore, CSCs surface PD-L1 could be regulated through protein modification such as ubiquitination and N-glycosylation 40, 41. In many CSC models, it has been identified that PD-L1 is involved in the immune evasion mechanisms of CSCs. In breast cancer, Wnt signaling pathway regulates co-expressing PD-L1 with CD44v6 and ALDH1A1, and promotes the expression of CD200 and CD276, which facilitates the immune escape 42. It was also reported that S100A14 inhibits STAT3-mediated PD-L1 expression, which negatively regulates colorectal cancer stemness and immune evasion 43. In addition, EMT has been acknowledged to drive the enrichment of PD-L1 in CSCs and promote immune evasion 7. These studies suggest complicated regulation mechanisms of CSC-intrinsic PD-L1 which warranties further investigation. Nevertheless, simultaneous targeting of CSC antigens as well as CSC signaling pathways while breaking down the immunosuppression may significantly enhance CSC-targeted immunotherapy.

## Crosstalk between CSCs and immunosuppressive cells in TME

**Figure 2:**
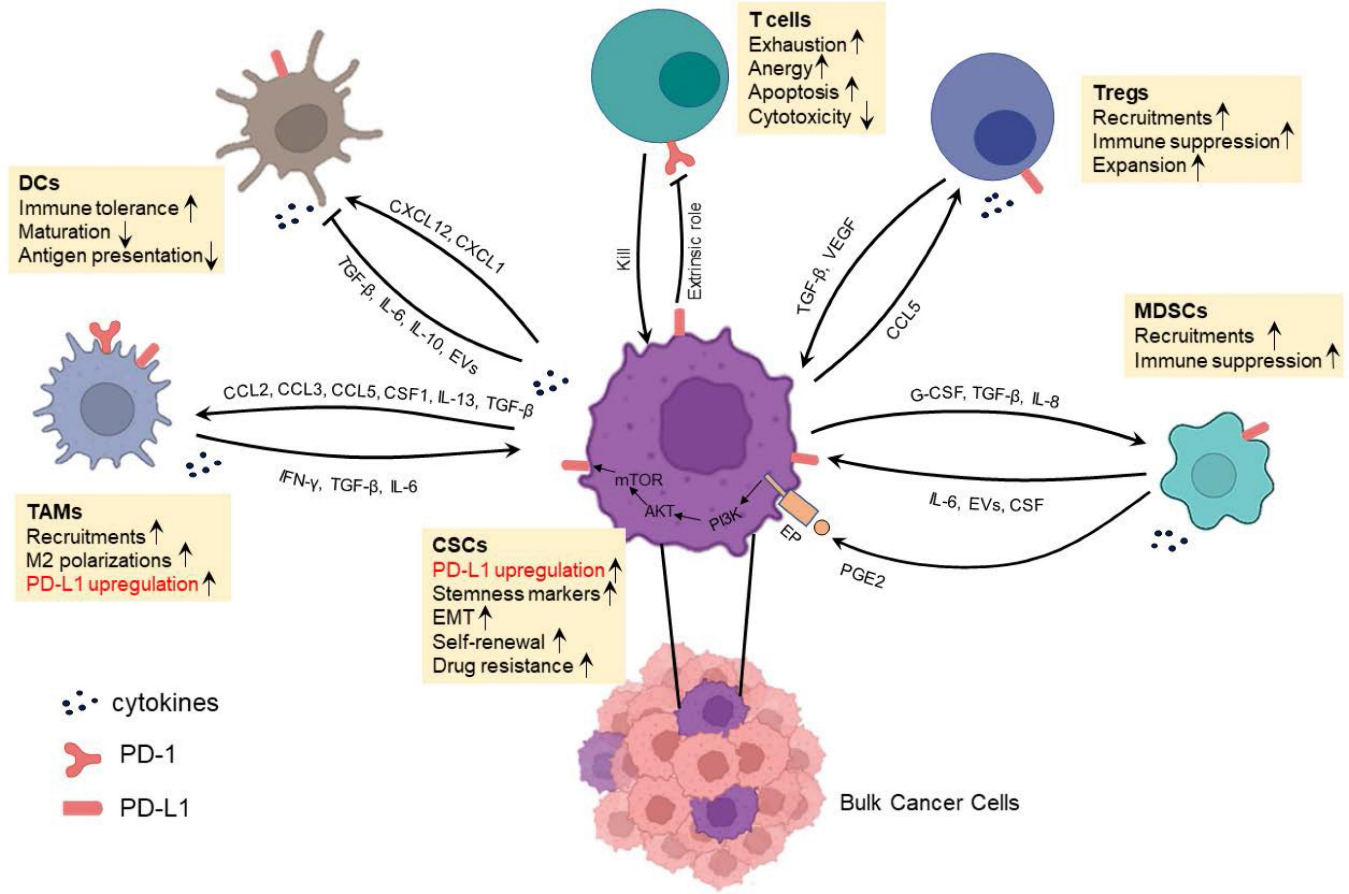
Crosstalk between CSCs and immune or immunosuppressive cells which involves PD-1/PD-L1 interaction in the immunosuppressive TME.

In addition to a direct binding between CSC surface PD-L1 and T cell surface PD-1 resulting in T cell exhaustion, anergy or apoptosis 44, CSCs also interact with immunosuppressive cells to achieve immune evasion and treatment resistance 45. CSCs actively interact with various types of cells as shown in **Figure 2** to induce their phenotype switching and function alternation, e.g., modulating the cytokine, chemokine secretion profiles. These immunosuppressive cells include DCregs, MDSCs, TAMs and Tregs. Recent studies have suggested that resistance to anti-PD-L1 therapy is in large part conferred by immunosuppressive cells. Of note, CSCs and these immunosuppressive cells express PD-1 and/or PD-L1 at various levels. In addition, MDSCs could induce the expression of PD-L1 in CSCs by secreting PGE2 followed by activating PI3K/AKT/mTOR pathway in CSCs. These phenomena usually result in the failure of antitumor effects of infiltrating T cells by extrinsic PD-1/PD-L1 axis thus adding rational to the simultaneous targeting of CSCs and the PD-1/PD-L1 axis in cancer immunotherapies. It is therefore important to understand the interaction between CSCs and immunosuppressive cells, particularly the impact of PD-L1 in these interactions.

### Interaction between CSCs and DCregs

DCs have been implicated as key regulators that guide responses to immune checkpoint blockers (ICBs) in cancer immunotherapies. Recent studies have shown the plasticity of DCs in TME that a subset of DCs may alter their roles to immunosuppressive at advanced stages of tumor progression, thus termed as DCregs 46. Moreover, there are growing appreciations of the protumor roles of reciprocal communications between CSCs and DCs. CSCs interfere with DC recruitment to the tumor site; impair their maturation and promote their differentiation into immunosuppressive DCs 23, 47, 48. Grange *et al.* reported that CD105^+^ renal CSCs are able to impede the differentiation of DCs from monocytes to a greater extent than CD105^-^ non-CSCs by a mechanism mainly related to the expression of human leukocyte antigen (HLA)-G released from extracellular vehicles (EVs) 48. Zhong *et al.* indicated that TGF-β/AKT/Smad2 signaling in human liver cancer plays a critical role in cancer stemness and in impairing CD86 and MHC-II expression on DCs, which resulted in immune tolerance 23.

Studies also shown that PD-L1 on the surface of DCregs in immunosuppressive TME induces T-cell dysfunction 49. Hence, anti-PD-L1 treatment blocking PD-L1 on both DCs and CSCs may explain the synergistic effects of DC vaccines combined with anti-PD-L1 treatment in mouse models 50, 51. However, whether the interaction between CSCs and DCregs involves a direct PD-1/PD-L1 engagement is unclear.

### Interaction between CSCs and MDSCs

MDSCs are a cluster of heterogeneous populations originated from myeloid cells with potent immunosuppressive effects and have emerged as important regulators of immune responses in cancers 52. Increasing evidence have revealed sophisticated interactions between CSCs and MDSCs in TME 53. In breast cancer, CSCs exhibit enhanced G-CSF production involving mTOR signaling and stimulate MDSC accumulation. In turn, MDSCs could increase CSC frequency through activating Notch in tumor cells, thus establishing a feed-forward loop 54. Moreover, in clinical specimens of breast cancer, the presence of MDSCs correlates with the existence of CSCs. Further investigations manifested an increase in the ALDH^high^ fraction with self-renewal capacity in human breast cancer cells after co-cultured with MDSCs, and the co-culture induced IL-6-dependent phosphorylation of STAT3 and activated Notch *via* nitric oxide (NO) in cancer cells 55. Furthermore, MDSCs increased STAT3 phosphorylation; CD133 and CD44 expression and sphere formation of mouse and human colorectal cancer cell lines* in vitro via* secretion of exosomal S100A9 56. Indeed, STAT3 activity seems to be a key mediator in the crosstalk between CSCs and MDSCs. In epithelial ovarian cancer, MDSCs enhanced the stemness by inducing the CSF2/p-STAT3 signaling pathway 57. Not only in solid cancers but in multiple myeloma, MDSCs endowed stemness qualities to malignant cells by inducing piRNA-823 expression and DNMT3B activation 58. In addition, CD133^+^ melanoma CSCs activated TGF-β1 expression through modulating miRNA-92 and recruited immunosuppressive MDSCs in the tumor site 59. Furthermore, glioblastoma patient-derived CSCs selectively drove MDSCs-mediated immune suppression by secreting high level of MIF which increased production of the immune-suppressive enzyme arginase-1 in MDSCs in a CXCR2-dependent manner 60.

Komura *et al.* 61 reported that co-culture MDSCs with ovarian cancer cells *in vitro* resulted in more ALDH^high^ CSCs and enhanced expression of PD-L1 in ALDH^high^ CSCs. This effect was achieved by activating the PI3K-AKT-mTOR pathway *via* the production of PGE2 by MDSCs. Treatment of ovarian cancer cells with rapamycin significantly inhibited this MDSC-mediated increase in PD-L1 expression. These studies demonstrate that MDSCs could induce PD-L1 expression in CSCs, suggesting that breaking the interaction between CSCs and MDSCs and/or blocking the PD-L1 expression in CSCs may help enhance CSC-targeted immunotherapy.

### Interaction between CSCs and TAMs

TAMs can be classified as antitumor M1 and protumor M2 subtypes which represent the extremes of a wide spectrum of differentiation states. Moreover, the abundance of M2 in tumors is usually associated with poor prognosis mainly due to their immunosuppressive functions. TAMs secrete several cytokines including IFN-γ, VEGFA, TGF-β and IL-6 that manifest key mediators of their immunosuppressive functions and foster CSC phenotypes 62, 63. CSCs recruit macrophages through the CC chemokines, the CXC chemokine subfamily, IL-33, and other soluble proteins 62. Moreover, CSCs may impact the polarization of TAMs to protumor M2 state by secreting elevated levels of CCL2, CCL5, CSF1, IL-13, TGF-β, periostin and CCN4 than their non-CSC counterparts 62. It was reviewed that various pro-inflammatory cytokines in TME, including IFN-γ, TGF-β, IL-6, IL-8, and IL-10 could upregulate the expression of PD-L1 and promote tumor progression 35. In particular, CSC-secreted such cytokines can induce the polarization of macrophages to M2 TAMs, suggesting that CSCs could influence the expression of PD-L1 in TAM polarity. On the other hand, TAMs promote CSC stemness through chemokines; soluble protein molecules and extracellular vesicles 62. For example, CCL5 derived from TAMs could promote prostate CSC self-renewal and cancer metastasis *via* activating β-catenin/STAT3 signaling 64. These highlight the complexities of CSC-TAM crosstalk and underlay the necessity of simultaneously targeting TAM-induced immunosuppression and stemness phenotypes. Importantly, TAMs express both PD-1 and PD-L1 increasingly over time as developing from bone marrow monocytes to mature macrophages and the PD-1 and PD-L1 expression further increases with disease progression after being recruited to tumor tissues 65. Zhu *et al.* observed that PD-1/PD-L1 could polarize TAMs to M2 phenotype 66. Liu *et al.* reported that high PD-L1 expression in TAMs is associated with the level of PD-L1 in both tumor cells and infiltrating CD8^+^ T cells 67. These studies suggest that breaking the interaction between CSCs and TAMs may benefit CSC-targeted immunotherapy.

### Interaction between CSCs and Tregs

Napoletano *et al.* observed a positive correlation between the presence of CSCs and Tregs in cancers 68. In general, Tregs are actively recruited into TME through various chemokines. Among different cancer types, diverse chemokines are preferentially secreted by CSCs *vs.* non-CSCs to attract Tregs. Sorted CD133^+^ ovarian CSCs showed elevated CCL5 production relative to non-CSCs. The interaction of CCL5 with its receptor CCR5 on Tregs in ovarian cancer patients resulted in Tregs recruitment. In return, recruited Tregs secret a higher level of IL-10 exerting pronounced immune-inhibitory function in CSC-enriched environments 69. SOX2-overexpressing breast cancer cells activated NF-κB-CCL1 signaling to recruit Tregs which in turn upregulated the stemness of breast cancer cells evident by increased ALDH^high^ population and enhanced stemness gene expression 70.

TGF-β is critical for the communication between CSCs and Tregs through several mechanisms. Firstly, high level of TGF-β secreted by Tregs directly promotes cancer cell stemness 71. Secondly, TGF-β indirectly participates in CSC formation and maintenance by inducing vascular endothelial growth factor (VEGF) which stimulates angiogenesis 72. Moreover, Tregs could promote the M2-polarization of TAMs through TGF-β signaling, further contributing to the immunosuppressive niche and CSC properties as described above 73.

Fortunato *et al.* reported that PD-L1^+^ CSCs in non-small cell lung cancer are able to specifically increase the percentage of Tregs in culture, and this effect could be abrogated by CXCR4 inhibitors 74. In accordance with these* in vitro* observations, inspections of clinical samples from metastatic lymph nodes of non-small cell lung cancer patients also proved the positive correlations of PD-L1^+^ CSCs with Tregs. One of the mechanisms underlying the negotiation between PD-L1^+^ CSCs and Tregs is that PD-L1 could augment Treg generation from naïve T cells by antagonizing the Akt-mTOR signaling cascade and thus enhance suppressive functions 75. Taken together, these studies suggest that breaking the interaction between CSCs and Tregs by blocking the PD-L1 signaling pathway may enhance the overall efficacy of immunotherapy against CSCs.

### Interactions between CSCs and NK cells and B cells

CSCs also interact with other immune cells in TME. Natural killer (NK) cells play a vital function in the immune system's defense against infections and tumor. Compared to normal cells, CSCs exhibit lower or no expression of MHC-I, which makes them more susceptible to recognition and elimination by NK cells 76. It was reported that NK cells could directly eliminate CSCs through receptor/ligand connections 77, 78. For example, CSCs express high levels of ULBP1, ULBP2, and MICA, which are NKG2D ligands 79, 80. The NKG2D-NKG2D ligand interaction is the primary pathway through which NK cells exert their antitumor effects on CSCs 52. In addition, NK cells can induce CSCs to differentiate into less-invasive and less-metastatic tumor cells 81. Although CSCs are susceptible to recognition and elimination by NK cells, they can induce NK cell exhaustion or block NK cell recognition by modulating the expression of NKG2D ligands; immune checkpoints and immunosuppressive cytokines such as PD-L1, TGF-β and IL-10, which facilitate CSC immune evasion 82, 83. The well-identified NKG2D ligands may serve as ideal markers in breaking immunosuppression to enhance CSC-targeted immunotherapy.

Tumor-infiltrating B cells have emerged as important players in cancer immunity and served as predictors of response to immunotherapy 84. Notably, not all B cells possess antitumor capabilities, and a subset of B cells, e.g., Bregs, are immunosuppressive. The research on the interplay between B cells and CSCs is relatively limited. It was demonstrated that the specific binding of IgG produced by CSC-DC vaccine-primed B cells could directly target and kill CSCs 85. However, other studies have indicated that a specific CSC-like B cell subpopulation exhibited self-renewal and multilineage differentiation capabilities in diffuse large B-cell lymphoma, which was regulated by *HMGB3*, *SAP30*, and* E2F8* 86. These findings suggest that the biological interaction between B cells and CSCs is a complex landscape, and warranties further investigation.

## Block PD-1/PD-L1 interruption to enhance CSC-targeted immunotherapy

The concept of CSC represents a novel direction in CSC-targeted immunotherapy. PD-1 is predominantly expressed on the surface of antigen-stimulated T cells and transduces inhibitory signals capable of restraining the activity of these cells, while PD-L1 can be expressed by various cell types, including professional antigen-presenting cells, cancer cells and CSCs. In addition to the immunomodulatory actions of PD-L1 in CSCs, tumor intrinsic PD-L1 could directly promote the maintenance of CSC stemness 30. Both CSCs and immunosuppressive cells express high levels of PD-L1 and they interact as reviewed in **Part 2.** This rationalizes the promises of combined anti-CSC strategy and PD-1/PD-L1 blockage in cancer immunotherapy.

### CSC-targeted vaccines

The immune evasion property of CSCs leads to failures of immunosurveillance. CSC-targeted DC vaccines aim to reverse the ignorance of the immune system to CSCs by loading CSC-antigens onto the DCs as a vaccine to induce CSC-specific T cells and B cells/antibodies which in turn effectively eliminate the CSC antigen-bearing CSCs.

Several groups, including our own, have employed DC vaccines to induce immunological recognition and toxicity towards CSCs. Pellegatta and colleagues 87 attempted to generate CSC-DC vaccine by pulsing the lysate of CSC-enriched neurospheres (NS) from murine GL261 glioma cells onto DCs (NS-DC). Compared to adherent cell lysate pulsed DC vaccines (AC-DC), NS-DC cured more tumor significantly. Later, Xu *et al.* used both human samples and a syngeneic animal brain tumor model to investigate the immune response of glioblastoma-derived CSC-DC vaccine. Vaccination of DCs loaded with CSC lysate induced Th1 immune response and achieved a significant survival benefit in the brain tumor model 88.

Our group sorted murine ALDH^high^ CSCs in two histologically different tumors (D5 melanoma and SCC7 squamous cell cancer) and the application of ALDH^high^ CSC lysate-DC vaccine resulted in direct targeting of CSCs by both cellular and humoral immunity 89, 90. Consistent findings have been obtained by designing CSC-DC vaccines by other CSC markers such as CD105, CD24/CD44 91, 92.

**Figure 3:**
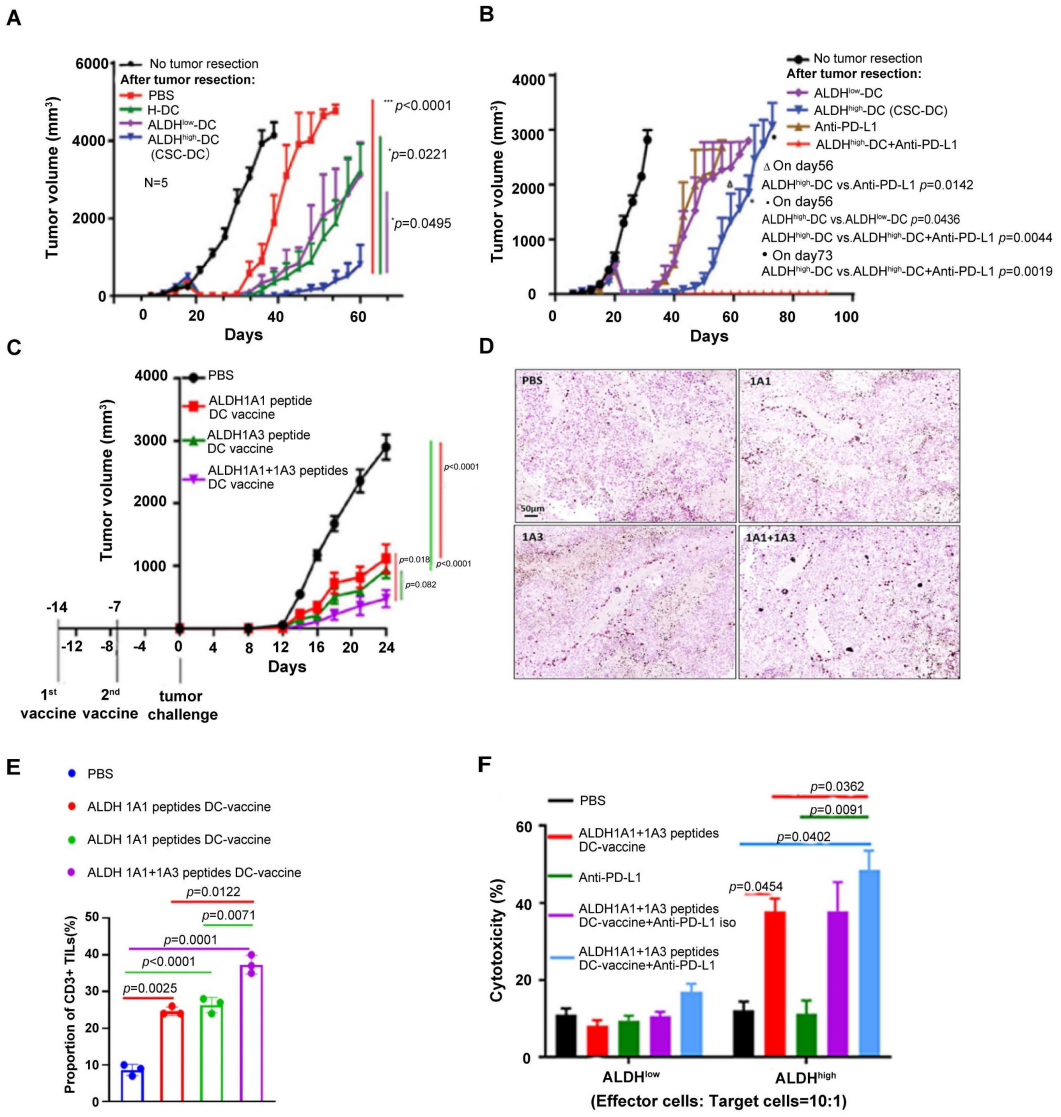
ALDH^high^ CSC lysate-DC and ALDH peptides-DC vaccination with anti-PD-L1 therapy significantly inhibited cancer growth. (**A**) Vaccination of DCs pulsed with the lysate of ALDH^high^ SCC7 CSCs (CSC-DC) significantly delayed tumor recurrence. Sourced from 93. (**B**) Administration of anti-PD-L1 further significantly inhibited tumor recurrence in animals treated with ALDH^high^ SCC7 CSC-DC vaccination (ALDH^high^-DC + Anti-PD-L1) after surgical resection of the subcutaneous SCC7 tumors as in (**A**). Animals remined tumor-free till day 90 when the experiment was terminated. Sourced from 93, reproduced with permission from American Association for Cancer Research publisher. (**C**) ALDH1A1+ALDH1A3 peptides-DC vaccine inhibited D5 tumor growth significantly more than single ALDH peptide-DC vaccine. Sourced from 98. (**D**) ALDH peptide-DC vaccination treatment induced CD3^+^ TILs. Representative immunohistochemical images show CD3^+^ TILs in residual tumors harvested from the treated hosts. Sourced from 98 (**E**) Bar graph comparing the CD3^+^ TILs induced by different treatments as indicated. Sourced from 98. (**F**) Cytotoxicity of splenic T cells isolated from D5-bearing mice treated in (**C**) (E/T = 10:1 ratio) against D5 ALDH^high^ CSCs vs. ALDH^low^ non-CSCs. Sourced from 98, reproduced with permission from Springer Nature publisher.

In a following up study in adjuvant setting, we found that the ALDH^high^ CSC-DC vaccine significantly delayed tumor recurrence, resulting in significantly prolonged animal survival after surgical resection of the subcutaneous tumors (**Figure 3A**) 93. Importantly, we found that ALDH^high^ CSC-DC vaccination plus anti-PD-L1 administration significantly inhibited tumor relapse (**Figure 3B**) and further prolonged animal survival 93. Further work in our group administrated ALDH^high^ CSC-DC vaccine with both anti-PD-L1 and anti-CTLA-4. These triple therapies exerted more significant anti-melanoma immune effects than either CSC-DC vaccine alone or anti-PD-L1 plus anti-CTLA-4 94. These experiments clearly demonstrate that immunologically targeting CSCs with simultaneously blocking of the immunosuppressive components has the potential to significantly enhance the efficacy of CSC-targeted immunotherapy.

Although preclinical models have endowed CSC lysate-DC vaccines with promising efficacy, the realistic plights remain in their clinical translation due to the difficulty to obtain adequate amounts of tumor tissues from each patient to make CSC lysate for vaccine preparation. To address this, CSC derived or associated proteins may represent an alternative source of CSC antigen. Integrin β4 (ITGB4) was identified as a receptor for the basement membrane protein laminin and involved in the regulation of CSCs in a variety of malignancies 95, 96. We synthesized murine ITGB4 (mITGB4) protein, pulsed it to DCs to generate mITGB4-DC vaccine in murine breast cancer and head and neck squamous cancer models. mITGB4-DC vaccination inhibited both local tumor growth and lung metastases in both models, and addition of anti-PD-L1 administration significantly enhanced the therapeutic effectiveness 97.

More recently, we utilized ALDH1A1 and ALDH1A3 peptides derived from ALDH1 isoform to prime DCs and generated ALDH peptide(s)-DC vaccine 98. ALDH1A1+1A3 dual peptides-DC vaccine mediated an additive anti-tumor effect compared to single peptide-DC vaccines in a D5 melanoma model (**Figure 3C**). PD-L1 blockade significantly enhanced ALDH^high^ CSC-targeted vaccination 98. This effect was associated with D5 ALDH^high^ CSC specific cytotoxic T lymphocyte (CTL) activity (**Figure 3D-F**).

**Figure 4:**
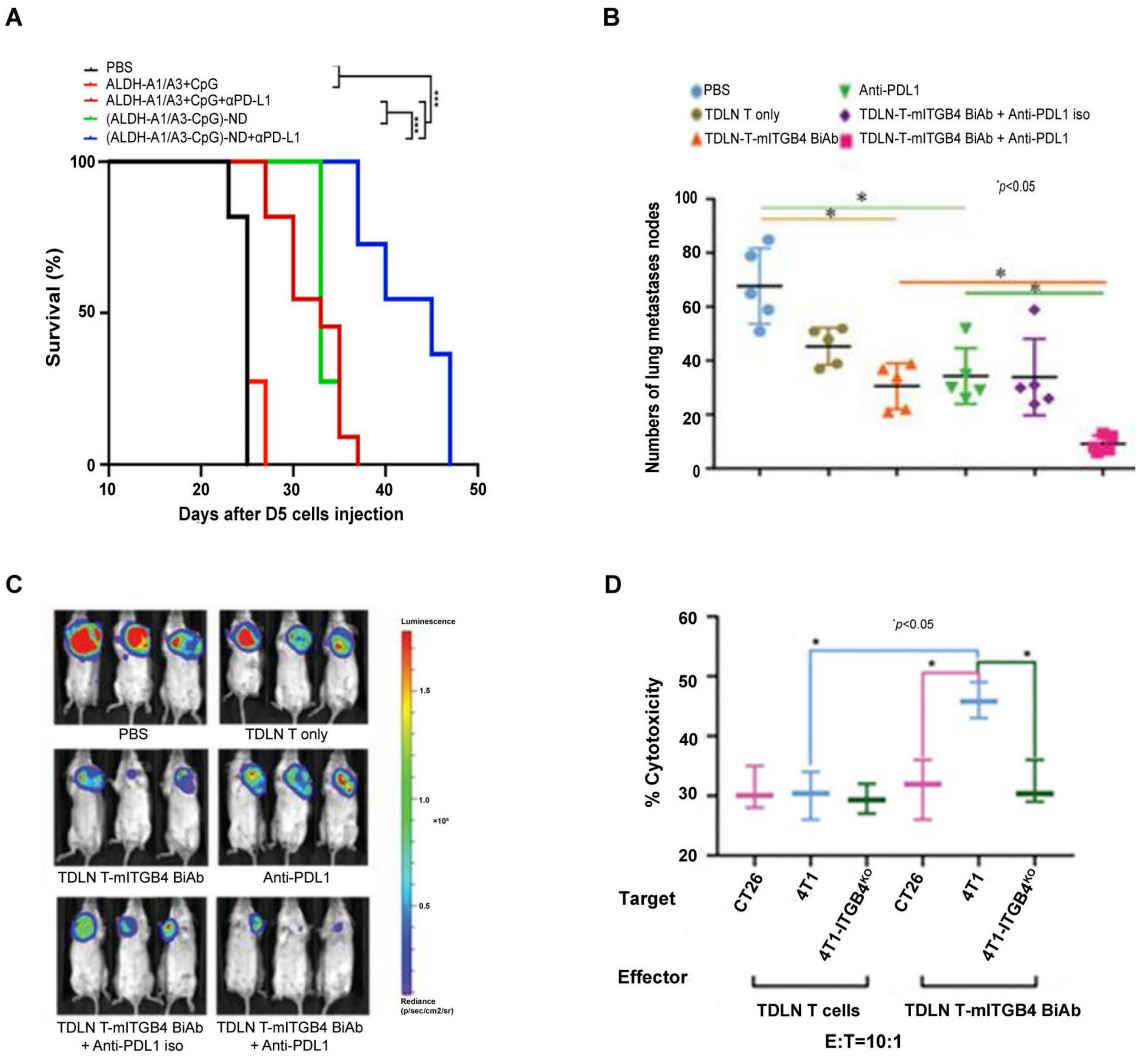
Anti-PD-L1 enhanced anti-CSC immunity induced by ALDH peptide-ND vaccine and mITGB4 BiAb-armed TDLN T cells. (**A**) Overall survival of C57BL/6 mice inoculated s.c. in the flank with D5 tumor cells and immunized with (ALDH-A1/A3-CpG)-ND nanoparticles or a soluble mixture of ALDH-A1/A3 and CpG (control). A subset of mice also received i.p. administration of anti-PD-L1 to enhance antitumor immunity. Sourced from 102. Copyright 2020 American Chemical Society. Reprinted with permission. (**B**) mITGB4 BiAb-TDLN T cells significantly suppressed the metastases in therapeutic 4T1 model. Source from 97. (**C**) mITGB4 BiAb-armed TDLN T cells significantly inhibited tumor growth in the therapeutic 4T1 model, which was enhanced by anti-PD-L1. Sourced from 97. (**D**) mITGB4 BiAb-armed TDLN T cells (TDLN T-mITGB4 BiAb) mediated greater cytotoxicity to ITGB4-expressing 4T1 cells than to 4T1-ITGB4 knockout (4T1-ITGB4^KO^) cells or ITGB4-negative CT26 cells *in vitro.* Sourced from 97, reproduced with permission from American Association for Cancer Research publisher.

To develop “off-the-shelf” CSC vaccines for cancer patients, we evaluated nano-vaccine systems in animal models 99. In initial studies, we co-loaded ALDH1A1 and/or ALDH1A3 peptides along with CpG (a TLR-9 agonist) to the synthetic high-density lipoprotein (sHDL) nanodiscs developed by our collaborator James Moon at the University of Michigan 100, 101. These nanodiscs efficiently delivered ALDH peptides to tumor-draining lymph nodes (TDLNs) and induced significant T cell responses against ALDH^high^ CSCs. Compared with soluble peptide vaccination, nanodiscs vaccination significantly slowed down tumor growth and prolonged the animal survival in D5 murine melanoma model. Of note, anti-PD-L1 therapy concurrently administrated with the ALDH peptide(s)-ND vaccine significantly enhanced the suppression on tumor growth and further prolonged the animal survival (**Figure 4A**) 102. This work represents the first attempt to developing an off-the-shelf nanoparticle-based vaccine strategy against CSCs to avoid the invasive DC collection from patients.

In our recent studies, we explored additional novel immunogenic peptide epitopes identified from CSC-associated transcription factors including SOX2 and NANOG. We synthesized two Sox2 and two Nanog-derived immunogenic peptides and co-loaded them along with the ALDH 1A1 and 1A3 peptides to the sHDL and formulated multi-CSC peptides-nanodisc cocktail vaccine 103. As a result, the multi-CSC peptides-nanodisc vaccine reduced tumor growth and extended animal survival significantly more than ALDH1A1/1A3 peptides-ND. We anticipate that immune suppression disruption, e.g., administration of anti-PD-L1 and/or anti-CTLA-4 may further augment the efficacy of this multi-CSC peptides-ND vaccine in CSC-targeted immunotherapy.

Taken together, CSC-targeted vaccines, based either on DCs or on NDs, have made breaking through progress in the past decade. The presentable CSC antigens have been optimized from CSC lysate to CSC associated proteins, and now to CSC marker protein-derived antigenic peptides. Multiple investigators, including us highlighted the benefits to block immunosuppressive signals to enhance CSC-targeted cellular and humoral immunity. This may lead to the development of novel immunotherapeutic regiment to treat cancer patient in clinic.

### Bispecific Ab targeting CSCs

Bispecific antibodies (BiAbs) against T cell markers and tumor cell markers bind to CD3 on T cells and antigens on tumor cells, resulting in the recruitment of T cells to the tumor. This is followed by T cell activation and degranulation and tumor cell elimination. T cell-engaging BiAbs have shown substantial effects in several hematological malignancies 104. However, T cell-engaging BiAbs in solid tumors have been less developed, partially due to the paucity of target molecules expressed on the solid tumor cell surfaces, which may lead to off-tumor toxicity. Catumaxomab (CD3-EpCAM BiAb) was the first approved T cell redirecting antibody for the treatment of malignant ascites 105, and it has been involved in clinic trials in several solid tumors including gastric cancer (NCT00464893), ovarian cancer (NCT01815528), and bladder cancer (NCT04819399). In attempt to specifically target CSCs, our group generated an anti-CD3/anti-CD133 BiAb that bound to effector cytokine-induced killer (CIK) cells (BiAb-CIK) to target CD133^high^ CSCs 106. In both mouse models of pancreatic and hepatic cancer, adoptive transfer of BiAb-CIK cells significantly inhibited CD133^high^ tumor growth than that by CIK cells or BiAb alone. On the other hand, resistance to BiAbs was observed in clinic, which was found at least partially due to the inhibitory effect on the T cell-tumor cell interaction through the PD-1/PD-L1 axis, leading to T cell dysfunction and exhaustion. Preclinical and clinical studies have shown that blockade of PD-1/PD-L1 axis benefits the antitumor activity of T cell-redirecting BiAbs 107, 108. In our effort to target ITGB4^high^ CSCs, we synthesized BiAb using anti-mITGB4 and anti-CD3 monoclonal antibodies and utilized it to arm TDLN T cells and generated mITGB4 BiAb-TDLN T cells. When transferred into 4T1 or SCC7 tumor-bearing mouse hosts 97, these T cells specifically targeted ITGB4^high^ CSCs and conferred host anti-CSC immunity, resulting in significant inhibition of local tumor growth and lung metastases, and this effect was significantly boosted by co-administration of anti-PD-L1 (**Figure 4B-D**).

### Antibody drug conjugate (ADC) targeting CSCs

**Figure 5:**
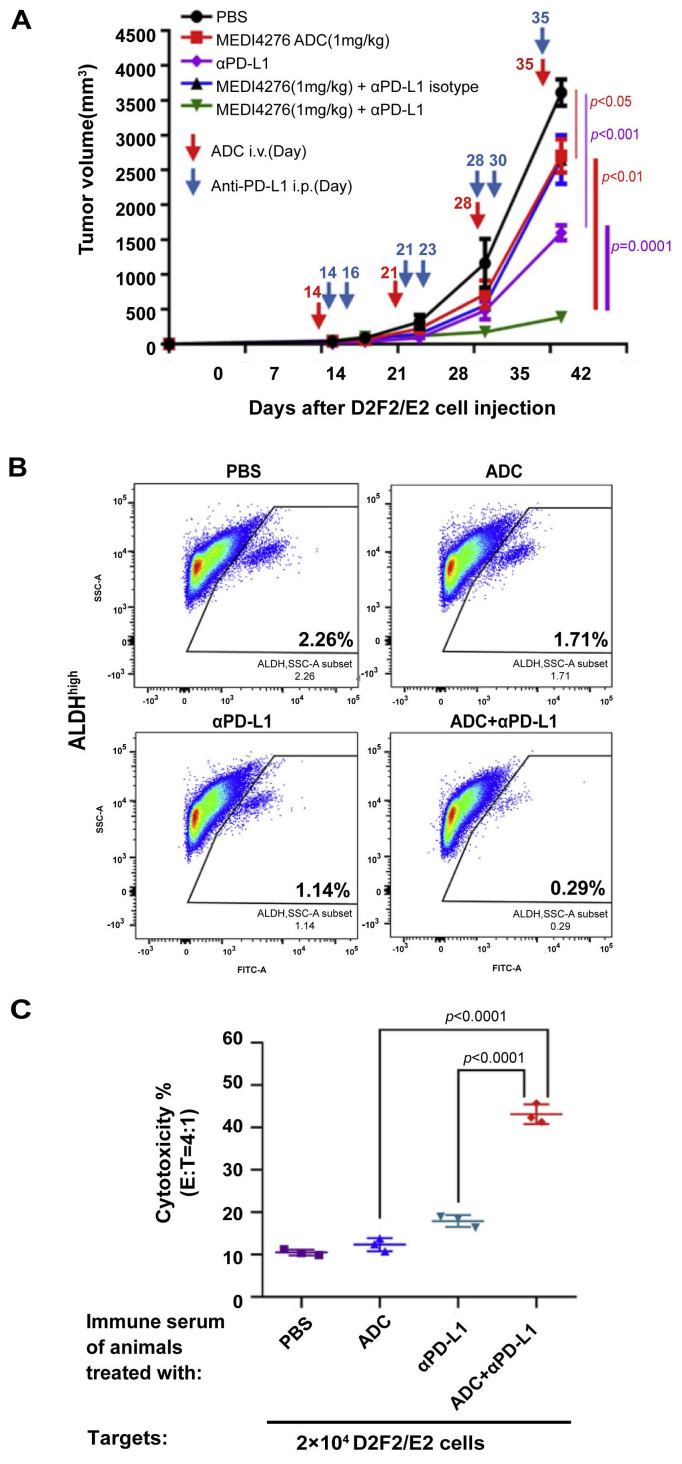
PD-l/PD-L1 blockade benefited HER2-targeted ADC immunotherapy to induce host immunity against CSCs. (**A**) Anti-PD-L1 significantly enhanced the efficacy of HER2-targeted ADC inhibiting D2F2/E2 tumor growth. (**B**) Evaluation of ALDH^high^ D2F2/E2 CSC frequencies in residual tumors after indicated treatment. (**C**) HER2-targeted ADC and anti-PD-L1 therapy induced significant humoral immunity against HER2-expressing tumor cells. Antibody-dependent cellular cytotoxicity (ADCC) assays were performed using the immune serum collected from the animals subjected to treatment as indicated to test IgG-mediated cytotoxicity against D2F2/E2 tumor cells. Source from 110, reproduced with permission from Elsevier publisher.

Antibody drug conjugate (ADC) represents an effective combination of target specificity and toxicity *via* the unique structure consisting of antibody (or antibody fragment), chemical linker, and cytotoxic payload. Human epidermal growth factor receptor 2 (HER2) has been one of the pivotal hotspots in ADC targets for its overexpression contributing to tumorigenic growth and CSC population increase in breast cancer 109. We tested MEDI4276 ADC against engineered human HER2-expressing murine breast cancers D2F2/E2 and HER2-4T1 in immunocompetent mouse models 110. MEDI4276 ADC demonstrated effective and specific anti-tumor activity in HER2-expressing cancer models *in vivo*, and anti-PD-L1 therapy significantly augmented the therapeutic efficacy of MEDI4276 ADC (**Figure 5A**). Importantly, these effects are associated with significant targeting of HER2^+^ALDH^high^ CSCs, resulting in reduction of this cell subset by ~90% post ADC + anti-PD-L1 therapy *vs.* PBS control (**Figure 5B**). Co-administration of anti-PD-L1 and MEDI4276 ADC significantly increased anti-tumor immunity of tumor-infiltrating lymphocytes (TILs) and host splenocytes. Furthermore, we identified HER2-targeted ADC treatment induced humoral immunity (**Figure 5C**) as well as T cell anti-tumor activity. These studies have demonstrated the benefits of anti-PD-L1 in ADC immunotherapy targeting CSCs.

### CSC-targeted chimeric antigen receptor (CAR)-T cell therapy

CAR-T cells are engineered T cells which express an artificial receptor specific for TAAs, leading to TAA-specific targeting and killing of cancer cells. CSC surface markers including CD44, EpCAM, CD47, CD123, CD133, TRKB have been reported as targets in CAR-T therapies in preclinical studies, and some of them are recently tested in clinical trials 111-115. A preclinical study indicated that anti-CD133 CAR-T cells exhibited pronounced killing efficiency of cisplatin-exposed CD133^+^ gastric cancer cells. Moreover, cisplatin and anti-CD133 CAR-T combination treatment inhibited gastric cancer progression with diminished CD133^+^ stem cell-like cell infiltration in mouse models 114. Phase I/II clinical trial (NCT02541370) has reported feasibility, controllable toxicities, and effective activity of CD133-directed CAR-T in patients with CD133^+^ hepatocellular carcinoma, pancreatic carcinomas, and colorectal carcinomas 116, 117.

Oncolytic adenovirus constructed to express a PD-L1 blocking mini-antibody successfully blocked the interactions between PD-1 and PD-L1, and increased the killing effect of HER2 CAR-T cells *in vitro,* and co-administration of the oncolytic adenovirus with HER2 CAR-T cells into xenograft prostate cancer models prolonged survival 118. More recently, Yamaguchi *et al.* demonstrated that PD-L1 blockade during the CD19 CAR-T cell transfer altered the M2 macrophages to more M1 like subsets, thus indirectly improved the antitumor activities of the CAR-T cells 119.

Collectively, these studies have suggested that CAR-T cells can be generated to mediate CSC-specific targeting, and this effort can be modulated by concurrent immunosuppression blockage. This may help develop more potent CAR-T cell therapy both for hematologic malignancies and for solid tumors.

### CSC-targeted NK cell therapy

NK cells simultaneously express inhibitory and activating receptors maintaining the subtle balance of transmitted signals after encountering target cells. These inhibitory receptors recognize various forms of MHC-I molecules on target cells 120. Reduction of MHC-I molecules on CSCs leads to more sensitivity to NK cells 77, 121, 122. Additionally, CSCs express higher levels of the ligands for NK cell activating receptors (such as NKG2D, NKp44 and NKp30) compared to non-CSCs 123-127. In this context, NK cell-mediated killing represents a promising approach for targeting CSCs 76.

Correspondingly, experiments on human breast, colon, melanoma, and glioblastoma revealed that NK cells could identify and eliminate CSCs in solid tumors 79, 128-130. Preclinical studies demonstrated that several CSC-specific antigens (e.g., GD2, HER2, CD133, PSCA, CLDN6)-targeted CAR-NK cells displayed superior anti-tumor activity 131-136. For example, CLDN6 was deemed as related to cancer stemness 136, and Li *et al.* recently reported that CLDN6 targeted CAR-NK cells could specifically kill CLDN6^+^ ovarian cancer cells *in vitro* 137*.* Furthermore, CLDN6 targeted CAR-NK cells successfully eliminated ovarian cancer cells in subcutaneous and intraperitoneal tumor models. However, CSCs can evade NK cell-mediated killing through interactions with prominent inhibitory receptors on NK cells such as PD-1, NKG2A, and KIRs 83, 138, 139. Rationally, combining ICBs with NK cell-based therapy may enhance anti-CSC responses. In this case, Li *et al.* found that CLDN6-targeted CAR-NK cells induced PD-L1 expression on the surface of tumor cells, and these PD-L1^+^ tumor cells were resistant to CAR-NK cells. More importantly, they revealed that combined with anti-PD-L1 synergistically enhanced the antitumor efficacy of CLDN6-targeted CAR-NK cells 137.

### Targeting CSC associated signaling pathways

Well-characterized signaling pathways that regulate the maintenance and survival of CSCs have proved as effective targets for CSCs, such as Hedgehog (Hh), Wnt/β-catenin, TGF-β and Notch pathways 140. For instance, Hh pathway is activated in basal cell carcinoma, gastrointestinal tract cancer, prostate cancer, breast cancer, glioblastoma, leukemia, and myeloma 141. Several Hh inhibitors were tested in different cancer types in clinic trials. Two FDA approved orally agents, vismodegib (GDC-0449) and sonidegib (LDE225), have demonstrated remarkable efficacies in locally advanced and metastatic basal cell carcinoma 142, 143. An ongoing clinical trial combining vismodegib with anti-PD-L1 administration (NCT05538091) is promising by concurrent immunosuppression blockage. The canonical Wnt/β-catenin pathway regulates stem cell pluripotency and determines the fate of cell differentiation. Correspondingly, PRI-724, an inhibitor of β-catenin, reduced drug resistance and CSC phenotypes in triple-negative breast cancer and downregulated SOX2 and CD44 expression in preclinical settings 144, 145. In addition, Osawa *et al.* described that combining PRI-724 with anti-PD-L1 treatment resulted in regression of tumor growth in a mouse model of colon cancer and provoked more profound antitumor CD8^+^ T cell response compared with each monotherapy 146. TGF-β pathway inhibitors have also been actively developed 147, 148. It is reasonable to anticipate that dual blockade of TGF-β and PD-L1 with bifunctional fusion proteins targeting TGF-β and PD-L1 149, 150, or co-administration of TGF-β receptor inhibitor galunisertib with the PD-L1 antibody durvalumab 151 will lead to more effective breaking down of the host immunosuppression, thus enhancing cancer immunotherapy targeting CSC-associated signaling pathways.

### Challenges and future prospects

Recently, the term “CSC plasticity” was proposed, which refers to the ability of these cells to switch between stem-like and differentiated states 152. For example, remarkable plasticity in the intestine has been found that various cell types can dedifferentiate into Lgr5^+^ cells to replenish the stem cell pool during perturbations to stem cells, thus acting as a hindrance to CSC-targeting therapy 153. In the current CSC-targeted immunotherapy, the majority of studies have identified CSCs based on established stem cell markers including ALDH, CD105, CD24, CD44, CD133, CD47, SOX2 and NANOG. However, this approach may increase the risk with off-target effects in CSC-targeting therapy, given the similarities in cell surface markers and stemness programs between adult stem cells and CSCs. In addition, CSCs exhibited significant resistance to chemotherapy; molecularly targeted therapy, and immunotherapy. CSCs require multiple mechanisms to escape immune surveillance. It was shown that CSCs with elevated EMT programs exhibit resistance to T cell cytotoxicity 154. Wnt/β-catenin stemness signaling in melanoma hinders the attraction of CD103^+^ dendritic cells, resulting in reduced T cell infiltration 155. Alternatively, CSCs can elevate the level of inhibitory receptors, e.g., PD-L1, CD47, and CD206, to evade immune response 152. Together, these challenges have suggested the limitations of translating CSC-targeted therapy to clinical applications for cancer patient treatment.

Given the immense heterogeneity and the plasticity of CSCs, accurate targeting of intrinsical CSC subset in tumor progress needs to be addressed. As single-cell profiling technologies advance in genomic, proteomic, and metabolic realms, coupled with spatial information 156, 157, they may provide hopeful approaches to discern the complex evolutionary changes of CSCs during the development of cancer. To address the immune evasion mechanisms of CSCs, research efforts may focus on pinpointing and targeting of molecular mechanisms that orchestrate the immune evasion of CSCs. As reviewed above, CSCs interact with the immunosuppressive cells in TME, such as DCregs, MDSCs, TAMs, Tregs, and Bregs to achieve immune evasion and diminish the efficacy of CSC-targeted immunotherapy. Destroying these interactions may help augment the effectiveness of such immunotherapy. For example, combination of oncolytic adenoviruses with anti-PD-L1 and anti-CTLA-4 synergistically enhanced the antitumor effect by recruiting CD8^+^ T cells and memory T cells, reducing the number of regulatory T cells, and promoting the polarization of TAMs from the M2 to the M1 phenotype 158. This strongly suggests that CSC-targeted immunotherapy with concurrent blockage of the immunosuppressive TME may represent an avenue to enhance the effectiveness to eliminate CSCs in clinic.

Several clinical trials and case studies targeting CSCs are summarized in **Table 2.** A phase I/II Trial of CSC-derived mRNA-transfected DC vaccine in glioblastoma patients showed that vaccination against CSCs was safe, well-tolerated, and prolonged progression-free survival (NCT00846456) 159. In another phase I study using lysate derived from an allogeneic GBM stem-like cell line to pulse autologous DCs was safe and well tolerated 160. However, additional challenges remain in the translation of this strategy. For instance, which tumor antigen source triggers the most potent immune response? We have reported that ALDH^high^ CSC lysis-DC vaccine 89, integrin β4 (a protein marker of CSCs)-DC vaccine 97, and ALDH peptide(s)-DC vaccine 98 respectively induced significant antitumor immunity. In recent years, several novel PD-L1 inhibitors including antibodies, small molecule inhibitors, and bifunctional small molecules are patented 35. The selection of the most appropriate PD-L1 inhibitor provides the opportunity and remains a challenge as well. Addressing these issues is crucial in future endeavors for the clinic practice to break immunosuppression to enhance CSC-targeted immunotherapy.

Indeed, new therapeutic approaches are being explored day by day. These include oncolytic viruses and cancer nanomedicine. Increasing ongoing pre-clinical trials demonstrated that oncolytic viruses could successfully abrogate CSCs 161-163. It was also reported that PD-L1 promotes oncolytic virus infection *via* a metabolic shift that inhibits the type I IFN pathway 164. This suggested the facilitate to explore the PD-1/PD-L1 interaction to modulate the oncolytic virotherapy. In addition, with the rapid development of nanotechnology, novel drug delivery systems specifically designed to target CSCs are on the rise. It was reported that CSC-targeted nanomedicine revealed the potential to overcome stemness-associated chemoresistance by multiple strategies, e.g. nanomedicine with ligand modification by polysaccharide, peptide, antibody or aptamer; co-loading the nano particles with chemotherapeutic and chemopotentiators, such as CSC-eliminating agent, chemosensitizer, self-renewal inhibitor, and differentiation-inducing agent 165-168. However, efficacious and unique stimuli for clinical practice with these encouraging novel strategies remain to be further explored.

## Conclusion

CSCs are heterogenous and plastic in different cancer types for disease initiation, progression, and relapse by escaping from immune surveillance and creating immunosuppressive microenvironment. Nevertheless, multiple anti-CSC immunological strategies have been developed including CSC specific vaccines, BiAbs, ADCs, and CAR-T cells as well as inhibitors for CSC associated signaling pathways. Accumulating literatures, including our own work, demonstrated that immune checkpoint blockade during these CSC-targeted immunotherapies could achieve more potent effectiveness. Despite this, more efforts are needed to improve our grasp over the intact roles of PD-L1 in CSCs, their involvement in the interactions with TME, and additional and more potent strategies to block the immunosuppression, which may pave the road for more precise development and utilizations of immune checkpoint blockades along with CSC-directed treatment in clinic.

## Figures and Tables

**Table 1:** Higher levels of PD-L1 expression in CSCs than in non-CSCs.

Cancer Type	Identification of CSCs by molecular markers	PD-L1 expression			Ref.
		CSCs	Non-CSSs	Detection Method	
Hepatoblastoma	CD34, CD90, OV-6, Vimentin	Positive	Negative	FC	5
Lung Adenocarcinoma	CD44	High	Low	IHC	9
Pancreatic Cancer	CD44, CD133	High	Low	IHC	11
Gastric Cancer	CD44, ALDH	High	Low	FC/WB	24
Breast and Colon Cancer	CD44, ALDH	High	Low	FC	26
Melanoma	ALDH	Positive	Negative	FC	27
Endometrial Cancer	CD133, ALDH, OCT4, SOX2, NANOG	High	Low	FC/WB/IF	28
Malignant Pleural Mesothelioma	ALCAM	High	Low	IHC	30
Ovarian Cancer	CD44, LGR5	High	Low	IHC	31

**Table 2:** Clinical Trials Targeting CSCs with Immunotherapies.

NCT number/ Case	Agent	Target	Cancer Type	Phase (N)	Primary endpoint	Results	Ref.
NCT00846456	DC vaccine with mRNA from GSCs	GSCs	Glioblastoma	Phase I/II (n=20)	AEs	Safe and well tolerated; prolonged PFS vs. controls	159
NCT02010606	DC vaccine pulsed with GSC lysate	GSCs	Newly diagnosed/ recurrent Glioblastoma	Phase I (n=36)	AEs	Safe and well tolerated; improved PFS/OS vs. controls	169
NCT01189968	Demcizumab + standard chemotherapy	CSCs	Metastatic non- squamous NSCLC	Phase I (n=46)	MTD	safe and well tolerated; 50% response rate	170
NCT03113643	SL-401 + Azacitidine or Azacitidine/ Venetoclax	CSCs	AML, BPDCN, high-risk MDS	Phase I (n=72)	MTD	Safe; 69% response rate	171
NCT02074046	Pancreatic CSC vaccine	CSCs	Pancreatic cancer	Phase I/II (n=90)	AEs	Safe and well tolerated; effectively activated CSC immune responses	172
NCT03030612	Cusatuzumab	LSCs	Newly diagnosed AML, high risk MDS	Phase I/II (n=12)	Toxicity; ORR	ORR=100%, reduced LSC number	173
NCT03222674	Anti-CLL1 CAR-T cells	LSCs	Pediatric R/R-AML	Phase I/II (n=8)	Treatment response; safety and tolerability	Well-tolerated; high targeting efficacy	174
Case	Anti -CLL1 CAR-T cells	LSCs	Secondary AML	n=1	Therapeutic response; MRD; CRS	CR> 10 months	175
Case	CART123 + Haplo-HSCT	LSCs	FUS-ERG^+^ AML, post-allo-HSCT relapse	n=1	Treatment response; CAR-T expansion; toxicity	Reduced AML blasts; full donor chimerism; myeloid implantation.	176

GSCs: Glioma stem cells; AE: adverse event; PFS: Progression-free survival; OS: Overall survival; NSCLC: Non-Small Cell Lung Cancer; MTD: Maximum tolerated dose; AML: Acute myeloid leukemia; BPDCN: Blastic plasmacytoid dendritic cell neoplasm; MDS: Myelodysplastic syndrome; LSCs: Leukemic stem cells; ORR: Overall response rate; R/R-AML: Relapsed/Refractory Acute Myeloid Leukemia; CLL1:C-type lectin-like molecule 1; MRD: Minimal residual disease; CRS: Cytokine release syndrome; CR: Complete remission; Haplo-HSCT: Haploidentical hematopoietic stem cell transplantation; FUS-ERG^+^ AML: Fused in Sarcoma and Erythroblast Transformation-Specific Acute Myeloid Leukemia; allo-HSCT: Allogeneic Hematopoietic Stem Cell Transplantation.

## Abbreviations

CSCs: cancer stem cells
PD-L1: programmed cell death ligand-1
ALDH: aldehyde dehydrogenase
Hh: hedgehog
NK: natural killer
MDSCs: myeloid-derived suppressor cells
TAMs: tumor-associated macrophages
DCregs: regulatory dendritic cells
Tregs: regulatory T cells
MHC: major histocompatibility complex
TAAs: tumor associated antigens
TGF-β: transforming growth factor β
EMT: epithelial-mesenchymal transition
UTR: untranslated regions
DCs: dendritic cells
ICBs: immune checkpoint blockers
HLA: human leukocyte antigen
EVs: extracellular vehicles
PMN: polymorphonuclear
NO: nitric oxide
VEGF: vascular endothelial growth factor
ITGB4: integrin β4
mITGB4: murine ITGB4
CTL: cytotoxic T lymphocyte
sHDL: synthetic high-density lipoprotein
CIK: cytokine-induced killer
TDLNs: tumor-draining lymph nodes
BiAbs: bispecific antibodies
HER2: human epidermal growth factor receptor 2
TILs: tumor-infiltrating lymphocytes
ADCs: antibody drug conjugates
CAR: chimeric antigen receptor
ADCC: antibody-dependent cellular cytotoxicity
